# Disentangling the temporal relationship between alcohol‐related attitudes and heavy episodic drinking in adolescents within a randomized controlled trial

**DOI:** 10.1111/add.16721

**Published:** 2024-12-10

**Authors:** Andrew Percy, R. Noah Padgett, Michael T. McKay, Jon C. Cole, Gregor Burkhart, Chloe Brennan, Harry R. Sumnall

**Affiliations:** ^1^ School of Social Sciences Education, and Social Work, Queens University Belfast UK; ^2^ Harvard T. H. Chan School of Public Health, Department of Epidemiology Harvard University Boston Massachusetts USA; ^3^ Northern Ireland Public Health Research Network, School of Medicine Ulster University Belfast UK; ^4^ Department of Psychology University of Liverpool Liverpool UK; ^5^ European Monitoring Centre for Drugs and Drug Addiction Lisbon Portugal; ^6^ Public Health Institute Liverpool John Moores University Liverpool UK

**Keywords:** Adolescents, alcohol, attitudes, autoregressive, cross‐lagged, drinking, random intercept, prevention, STAMPP, RCT, RI‐CLPM

## Abstract

**Background and aims:**

Within many alcohol prevention interventions, changes in alcohol‐related attitudes (ARA) are often proposed as precursors to changes in drinking behaviour. This study aimed to measure the longitudinal relationship between ARA and behaviour during the implementation of a large‐scale prevention trial.

**Design and setting:**

This study was a two‐arm school‐based clustered randomized controlled trial. A total of 105 schools in Northern Ireland and Scotland participated in the Steps Towards Alcohol Misuse Prevention Programme (STAMPP) Trial.

**Participants:**

A sample of 12 738 pupils (50% female; mean age = 12.5 years at baseline) self‐completed questionnaires on four occasions (T1–T4). The final data sweep (T4) was 33 months post baseline.

**Measurements:**

Individual assessments of ARA and heavy episodic drinking (HED) were made at each time‐point. Additional covariates included location, school type, school socio‐economic status and intervention arm. Estimated models examined the within‐individual autoregressive and cross‐lagged effects between ARA and HED across the four time‐points (Bayes estimator).

**Findings:**

All autoregressive effects were statistically significant for both ARA and HED across all time‐points. Past ARA predicted future ARA [e.g. ARA_T1_ → ARA_T2_ = 0.071, credibility interval (CI) = 0.043–0.099, *P* < 0.001, one‐tailed]. Similarly, past HED predicated future HED (e.g. HED_T1_ → HED_T2_ = 0.303, CI = 0.222–0.382, *P* < 0.001, one‐tailed). Autoregressive effects for HED were larger than those for ARA at all time‐points. In the cross‐lagged effects, past HED statistically significantly predicted more positive ARA in the future (e.g. HED_T2_ → ARA_T3_ = 0.125, CI = 0.078–0.173, *P* < 0.001, one tailed) except for the initial T1–T2 path. In contrast, past ARA did not predict future HED across any time‐points.

**Conclusions:**

Changes in alcohol‐related attitudes were not a precursor to changes in heavy episodic drinking within the Steps Towards Alcohol Misuse Prevention Programme (STAMPP) Trial in Scotland and Northern Ireland. Rather, alcohol‐related attitudes were more likely to reflect prior drinking status than predict future status. Heavy episodic drinking status appears to have a greater impact on future alcohol attitudes than attitudes do on future heavy episodic drinking.

## INTRODUCTION

A robust body of research suggests that an individual’s personal alcohol related attitudes (ARA) are important cognitive determinants of alcohol consumption, predicting both concurrent and prospective drinking behaviour [1, 2, 3, 4, 5]. More positive attitudes toward alcohol tend to be reported by individuals who drink more, with the reverse true for more negative attitudes [6, 7]. Drinking attitudes have also been identified as potent predictors of the quantity of consumption, drunkenness and heavy episodic drinking (HED) [5, 8, 9, 10], with drinking intentions tending to mediate the relationship between attitudes and consumption [2, 8, 11]. In general, attitudes towards alcohol among children and adolescents become more positive with age [12, 13, 14] and have been found to be associated with a range external factors, such as parental alcohol attitudes and consumption [15, 16], peer attitudes and consumption [11, 17], exposure to alcohol advertising [7] and alcohol‐related social media [18, 19].

To date, most studies in this area have tended to focus upon examining the relationship between ARA and drinking behaviours only at a between‐individual level, such as mean differences in attitudes across different drinking patterns, in the case of cross‐sectional studies (e.g. [20]) or have aggregated both within‐ and between‐individual changes in drinking or attitudes over time, in the case of longitudinal studies (e.g. [21]). In contrast, however, both relevant theory, such as the Theory of Planned Behaviour [8] and prevention practice (see, for example, [22]) postulates that the causal relationship between ARA and drinking behaviour occurs primarily at a within‐individual level, where an individual’s personal attitudes directly affect their own intentions and/or decision‐making. Failing to disentangle within‐individual from between‐individual effects may limit the ability to accurately quantify these effects and thus understand the causal relationship between ARA and drinking behaviour (see [23, 24] for further discussion of this issue).

Therefore, the present study aimed to examine the temporal bidirectional relationships between ARA and HED in a large sample of UK adolescent participants in a clustered randomized controlled trial (cRCT). The study utilized four waves of trial data. A multi‐level random intercept cross‐lagged panel model [25, 26] was estimated, utilizing a Bayes estimator [27], to disaggregate within‐individual changes in both primary processes (ARA and HED) over time from their stable trait‐like between‐individual differences (for example, stable differences between individuals in their propensity to heavy alcohol consumption). The study examined both the autoregressive relationships within each process (i.e. the extent to which current ARA predict future ARA) as well as cross‐lagged relationships (i.e. how current ARA may influence future HED and vice versa). It is the nature of these cross‐lagged relationships that are of particular importance to the design and delivery of primary prevention interventions aimed at reducing hazardous drinking in adolescents.

## METHODS

### Study design and participants

Data were from schoolchildren in 105 schools participating in a longitudinal cRCT examining the efficacy of a joint classroom and community‐based alcohol intervention [28, 29, 30]. The intervention was administered at school‐level, leading to a cRCT comparing the effects of the intervention (STAMPP) versus the alcohol education as normal among randomized schools. These data were gathered in schools in Northern Ireland (*n*_NI_ = 70) and Scotland (*n*_S_ = 35). Participating schools had an average of 121 trial participants, giving a total sample of 13 207 students. After accounting for students who did not respond to any items, the sample size reduced to 12 738. All data were collected using pencil‐and‐paper self‐report surveys of students completed in school. Completed questionnaires were optically scanned and quality assured by the Northern Ireland Clinical Trials Unit.

Prior to the start of data collection, opt‐in consent was obtained from school head‐teachers/principals. Similarly, opt‐out consent was obtained from participants and their parents/guardians. Data were collected under examination‐like conditions on school premises at baseline (T1, June 2012) and at three follow‐ups: +12 (T2), +24 (T3) and +33 (T4) months. For a more detailed account of the full trial methodology and data collection, please see the published study report [30]. The study was approved by the University Research Ethics Committee at Liverpool John Moores University.

### Measures

#### Heavy episodic drinking (HED)

HED was defined as the self‐reported number of occasions in the previous 30 days on which male students consumed ≥ 6 units of alcohol or female students consumed ≥ 4.5 units in a single episode. This frequency count was subsequently dichotomized at never (0) and one or more occasion (1). To improve the accuracy of self‐reported HED, students were provided with pictorial prompts of how much alcohol ≥ 6/≥ 4.5 UK units represented. The pictures presented the most popular drinks in amounts (i.e. number and size of bottles) representing ≥ 6/≥ 4.5 UK units. Given the gendered nature of the measure, the prompts were separately colour‐coded for male (showing 6 units) and for female students (showing 4.5 units). This measure was one of the two primary registered outcomes within the original cRCT, and the only outcome for which a successful intervention effect was detected [28, 29, 30]. A slightly different threshold for HED was used at baseline (T1). Here, HED was defined as consuming 5+ ‘drinks’ in the last 30 days, which was dichotomized into never (0) and once or more (1), as in later sweeps. This change was made with the full approval of the Trial Steering Committee [30] and prior to unblinding of the random allocation of schools to study arm and the initiation of any data analysis.

#### Alcohol‐related attitudes (ARA)

ARA were assessed by a six‐item scale [31]. A typical item was: ‘It is OK for young people to drink as long as they do it safely?’. Responses were scored using a five‐point Likert‐type scale ranging from strongly disagree to strongly agree. Three of the six items were reverse‐coded to ensure that higher scores equated to more positive attitudes towards alcohol. Parallel analysis confirmed a single‐factor structure. Full details of the items can be found in the Supporting information. Exploratory factor analysis (see Supporting information) suggested that two of the six items loaded weakly on the single factor. Models were estimated with both the six‐item scale and a revised four‐item scale (dropping the two lowest loading items). As no substantive differences were detected between the models using the four‐ or six‐item versions (see Supporting information), the original six‐item scale was used in the primary analysis.

#### Additional covariates

Additional variables included in the analyses were the intervention arm (control/intervention), school type (all boys, all girls or mixed gender), school socio‐economic status (SES) approximated using a tertile split of the proportion of students in receipt of free school meals (lowest third/middle third/highest third) and the location of the school (Northern Ireland/Scotland). These variables were all used in the randomization process within the cRCT and were included in the registered primary outcome analysis. In this study, the control variables were included in the analyses as time‐invariant covariates.

### Analysis strategy

For this study, we used an expanded version of the random‐intercept cross‐lagged panel model (RI‐CLPM [25,26]) to estimate the longitudinal reciprocal relationship between HED and ARA. The RI‐CLPM addresses some of the inherent weaknesses in standard autoregressive cross‐lagged models [25, 32, 33, 34]. Individual‐level random intercepts are used to capture the trait‐like components of both ARA and HED that vary between individuals but remain relatively stable, allowing the model to examine the interplay between ARA and HED within individuals over time (see [35, 36, 37] for comparisons of alternatives to standard autoregressive cross‐lagged models).

The RI‐CLPM used in this analysis was expanded to include additional random intercepts among schools. These school‐level random intercepts represent the common between‐person variance above and beyond individual differences (e.g. stable school‐level differences in drinking culture). The addition of school‐level random intercepts was our approach to account for the nesting of observations (pupils within schools). The factor loadings on the random intercepts (both individual level and school level) were constrained to one. The school level random intercepts also were regressed onto the time‐invariant covariates (intervention arm, school SES, school type and location), as these were all assessed at the school level.

Given that HED is a binary variable, standard maximum likelihood (ML) estimation cannot be used [27]. Therefore, a Bayes estimator was employed within the primary model to generate Bayesian posterior parameter estimates with credibility intervals (CI) (for full details see [38, 39, 40]). All models were estimated using Mplus version 8.8 [41]. Cases with missing on all variables were excluded from the analysis (*n* = 469). The remaining missing data were imputed using the Mplus Bayes estimation method, which combines the practical benefits of modelling categorical indicators with full information estimation.

#### Sensitivity tests

The primary model was also estimated using the WLSMV estimator to test the sensitivity of the model to the specific estimator used. In addition, models were estimated with different measures of adolescent alcohol consumption (frequency of drinking alcohol and personal experiences of alcohol‐related harms) in place of HED, to test the sensitivity of models to the measurement of drinking behaviour.

To test the sensitivity of the RI‐CLPM to misspecifications of the longitudinal mechanism, a range of additional models were estimated, including standard CLPM, models with additional lags, a model with constraints on the autoregressive and cross‐lagged effects and models with cluster robust standard errors accounting for clustering at the school, rather than school‐level random intercepts. Full details of the various models estimated and the different measures of attitudes and alcohol consumption can be found in the Supporting information. The full results of these additional specifications and sensitivity tests are available in our online resources (https://osf.io/bmhak/?view_only=67d2dc1013af46a880662a2c8067635a).

## RESULTS

### Sample characteristics

At the beginning of the study participants were in the single‐year group 8/S1 [mean_age_ = 12.5 years; standard deviation (SD)_age_ = 0.4 years]. Approximately 50% of the sample were female, 38% were from Scotland and 23% were in receipt of free school meals (an indicator of low SES). Almost all the sample were white (95%). Of the full sample, approximately 18% (*n* = 2333) were lost to follow‐up by T4. Schools were mainly mixed gender, with a small proportion being single‐sex schools (18%) (Table 1). Schools were randomized (1:1) to either the intervention arm or the control arm; two‐thirds were in Northern Ireland. While the proportion reporting HED increased among the four waves of data (8–21%), the average scores for ARA remained relatively stable over time (mean_ARA_ = 2.57–2.52). There was a higher rate of attrition among students who lived in Scotland (24%), were male (19%), in receipt of free school meals (26%) or had reported alcohol use at baseline (T1; 25%). The attrition rate was also slightly higher among those pupils in the control arm of the study (19%). At the school level, attrition rates varied from 1.5 to 32%. Full details of the study attrition can be found in the main trial report [30].

**TABLE 1:** Sample characteristics.

Sample characteristics	Students *n* (%)	Schools *n* (%)
Study arm		
Intervention	6379 (50.0)	53 (50.5)
Education as usual	6359 (50.0)	52 (49.5)
Location		
Northern Ireland	7742 (60.8)	70 (66.7)
Scotland	4996 (39.2)	35 (33.3)
Socio‐economic status		
Tertile 1	4288 (33.7)	33 (31.4)
Tertile 2	5012 (39.3)	40 (38.1)
Tertile 3	3438 (27.0)	32 (30.5)
School type		
Mixed boys and girls	10 481 (82.3)	87 (82.9)
All girls’ school	1224 (9.6)	10 (9.5)
All boys’ school	1033 (8.1)	8 (7.6)
Heavy episodic drinking^1^		
T1 (baseline)	863 (7.7)	
T2	940 (8.6)	
T3	1295 (12.4)	
T4	2179 (21.3)	
Attitudes towards alcohol, mean (SD)^2^		
T1 (baseline)	2.57 (0.44)	
T2	2.53 (0.44)	
T4	2.51 (0.45)	
T3	2.52 (0.44)	

^1^ Heavy episodic drinking (HED) percentage pupils missing, T1: 12.03; T2: 14.13; T3: 18.15; T4: 19.67;

^2^ attitudes percentage pupils missing, T1: 11.26; T2: 12.67; T3: 17.53; T4: 18.73. SD = standard deviation.

### Autoregressive and cross‐lag effects of heavy episodic drinking (HED) and alcohol‐related attitudes (ARA)

**FIGURE 1:**
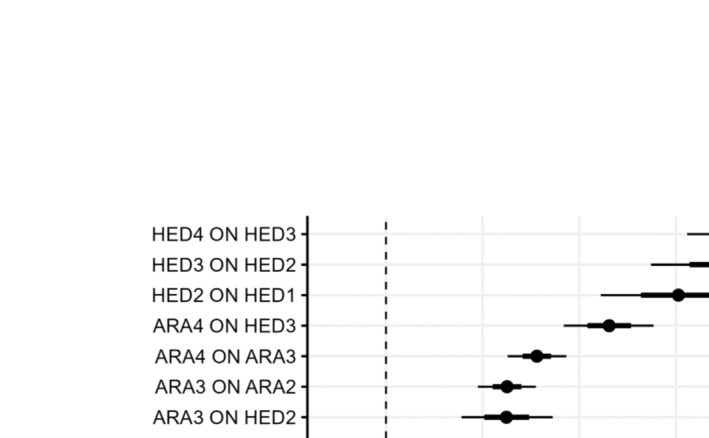
Heavy episodic drinking (HED) was significantly predictive of attitudes. Figure depicts the 95% central probability of posterior distributions

The posterior distribution of the autoregression and cross‐lag regression parameters are shown in Figure 1. The median of the posterior distribution of the standardized autoregression and cross‐lag regression parameters, together with their CIs, are displayed in Table 2. All autoregressive effects were significant for both HED and ARA. The median of the posterior distribution of the autoregressive effects also increased with time among the four data waves. For ARA, this increase equated to an approximate doubling of the autoregressive parameter estimates (from 0.07 to 0.16). Similarly, the size of the autoregressive parameters for HED also increased over time (from 0.30 to 0.39).

**TABLE 2:** Posterior distribution of standardized autoregressive and cross‐lag effects of heavy episodic drinking and attitudes related to alcohol.

Parameter	Median	SD	Q0.025	Q0.25	Q0.75	Q0.975	Sig	Rhat
Intercept correlation								
ARA ↔ HED	0.130	0.042	0.043	0.101	0.158	0.210	*	1.002
Autoregressive relationships								
ARA1 → ARA2	0.071	0.014	0.043	0.061	0.080	0.099	*	1.002
ARA2 → ARA3	0.125	0.015	0.095	0.115	0.136	0.155	*	1.001
ARA3 → ARA4	0.156	0.015	0.126	0.146	0.167	0.187	*	1.001
HED1 → HED2	0.303	0.041	0.222	0.276	0.330	0.382	*	1.005
HED2 → HED3	0.352	0.040	0.274	0.325	0.380	0.428	*	1.003
HED3 → HED4	0.389	0.038	0.312	0.363	0.414	0.463	*	1.004
Cross‐lag relationships								
HED1 → ARA2	0.033	0.024	−0.013	0.017	0.049	0.079		1.000
HED2 → ARA3	0.125	0.024	0.078	0.108	0.141	0.173	*	1.001
HED3 → ARA4	0.231	0.024	0.184	0.215	0.247	0.277	*	1.001
ARA1 → HED2	0.010	0.022	−0.032	−0.004	0.025	0.054		1.002
ARA2 → HED3	−0.003	0.022	−0.045	−0.018	0.011	0.039		1.003
ARA3 → HED4	−0.021	0.018	−0.055	−0.033	−0.009	0.014		1.001

*Note*: *n* = 12 738; Outcomes are the within‐person estimates of the longitudinal relationship between heavy episodic drinking (HED) and attitudes after accounting for school‐level differences. Single‐headed arrows represent regression slopes. For example, 1 → alcohol‐related attitudes 2 (ARA2) represents an autoregressive path where attitudes at T2 is regressed on attitudes at T1. Similarly, HED1 → ARA2 represents a cross‐lag path where attitudes at T2 was regressed onto HED at T1. Double‐headed arrows represent correlations. ARA ↔ HED is the correlation between the estimated intercepts for attitudes and HED. Median = median of posterior distribution; SD = standard deviation of posterior; Q0.X = Quantile of posterior distribution, e.g. 2.5% quantile or 97.5% quantile; Sig = indicator of whether the central 95% credible interval of posterior contains 0 or not; and Rhat = Gelman–Rubin–Brooks convergence criteria at the end of sampling posteriors. All time‐invariant predictors also included within the model. All reported estimates are the fully standardized salutation (STDYX).

Regarding the cross‐lag effects, past HED was observed to predict future ARA (except from baseline T1 to the first follow‐up T2). From T2 onwards, engagement in HED significantly predicted more positive attitudes towards alcohol in the future. In contrast, however, past attitudes did not predict future HED at any time‐point. It should be noted that the standardized cross‐lag effects (e.g. HED2 → ARA3 = 0.13) were generally smaller than those observed for the autoregressive effects (e.g. HED2 → HED3 = 0.35). However, the largest single effect on later ARA was prior HED at T3 (HED3 → ARA4 = 0.23, 95% CI = 0.18–0.28). Similarly, the largest influence on later HED was also prior HED at T3 (HED3 → HED4 = 0.39, CI = 0.31–0.46).

While sizeable differences in parameter estimates were detected among some of the sensitivity models estimated these were mainly restricted to comparisons of the primary multi‐level RI‐CLPM with standard CLPMs, or with models where cluster robust standard errors (SEs) were used to account for clustering at the school rather than school‐level random intercepts (see Supporting information, Table S2). Even here, the pattern of significant effects remained largely the same. When comparing the primary model to other multi‐level RI‐CLPMs employing different estimators or with different measures of alcohol consumption or attitudes, the differences were modest. While some parameters changed sign, this tended to occur only when the parameter in the primary model was already very close to zero.

### Time invariant predictor effects

**FIGURE 2:**
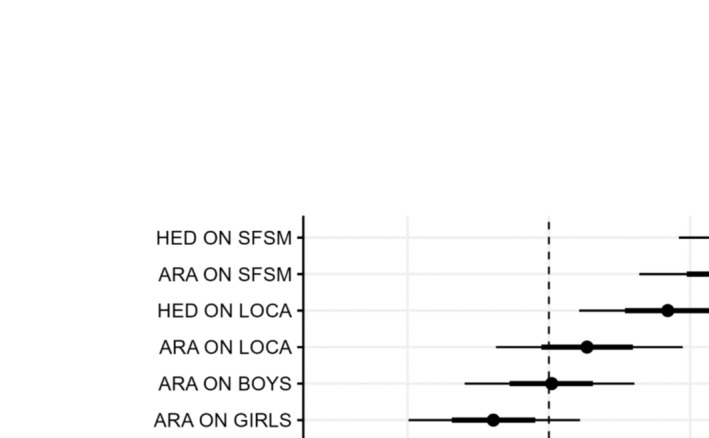
Time invariant covariates were stronger predictors of heavy episodic drinking (HED) than attitudes. SFSM = socio‐economic status (tertile split); LOCA = location of school; BOYS = all‐boys school; GIRLS = all‐girls school; and ARM = study arm

When examining the time‐invariant predictors, the full multi‐level RI‐CLPM provided evidence that the intervention was significantly related to lower school average ARA scores, but not a statistically significant decrease in the proportion of HED (see Figure 2 and Table 3). Lower school SES was similarly associated with more positive ARA. In contrast, higher rates of HED were observed in schools in Scotland, in schools with a lower SES and in mixed‐sex schools relative to single‐sex schools (either all‐boys schools or all‐girls schools).

**TABLE 3:** Posterior distribution of time‐invariant predictor standardized effects (HED‐ARA model).

Predictor	Median	SD	Q0.025	Q0.25	Q0.75	Q0.975	Sig	Rhat
Attitudes								
Study arm^1^	−0.216	0.087	−0.382	−0.275	−0.157	−0.039	*	1.000
Location^2^	0.081	0.101	−0.113	0.012	0.149	0.284		1.001
School SES^3^	0.382	0.091	0.192	0.320	0.441	0.545	*	1.001
Girls’ school^4^	−0.118	0.093	−0.298	−0.181	−0.055	0.066		1.000
Boys’ school^4^	0.006	0.092	−0.179	−0.058	0.067	0.182		1.000
HED								
Study arm^1^	−0.146	0.082	−0.304	−0.202	−0.091	0.016		1.001
Location^2^	0.252	0.095	0.064	0.188	0.316	0.438	*	1.000
School SES^3^	0.450	0.085	0.276	0.391	0.507	0.605	*	1.001
Girls’ school^4^	−0.306	0.085	−0.468	−0.364	−0.248	−0.135	*	1.000
Boys’ school^4^	−0.235	0.084	−0.395	−0.290	−0.177	−0.068	*	1.000

*Note*: *n* = 12 738; Outcomes are the school‐level average heavy episodic drinking (HED) and average alcohol‐related attitudes (ARA) scores. Median = median of posterior distribution; SD = standard deviation of posterior; Q0.X = quantile of posterior distribution, e.g. 2.5% quantile or 97.5% quantile; Sig = indicator of whether or not the posterior contains 0; and Rhat = Gelman–Rubin–Brooks convergence criteria at the end of sampling posteriors. 1 ref cat = control school; 2 ref cat = Northern Ireland; 3 tertile spilt on proportion of free schools meals; 4 ref cat = mixed‐gender school.

## DISCUSSION

The findings suggest a high degree of within‐individual consistency over time in both adolescent self‐reported ARA and HED. Prior attitudes and prior drinking behaviours were the main predictors of future attitudes and behaviours, respectively. Of all the paths tested, it was the autoregressive paths (ARA_t_ → ARA_t + 1_; HED_t_ → HED_t + 1_, etc.) that generally had the strongest longitudinal effects. The one exception to this was the influence of HED at T3, which had the largest single effect on future ARA of any of the autoregressive or cross‐lagged predictors.

At the sample level, mean ARA remained relatively constant over time, although this undoubtedly masks a degree of individual variation. As participants aged, the influence of prior attitudes on future attitudes increased in line with most psychological theories of learning (e.g. [2]). While there is inherent growth in the rate of HED over time, most students maintained the same HED status during the 4 years of the study (i.e. remained within the non‐HED group). Even when students made a transition into HED, they were also likely to remain consistent in this newly acquired behaviour in future. This long‐term consistency in adolescent drinking is well documented [42, 43, 44, 45]. Even among emergent adolescent drinkers, alcohol consumption can be thought of as both a state and a trait [46], where the stable trait for risky drinking is largely determined by dispositional decision‐making characteristics, while state fluctuations in drinking were largely driven by more situational decision‐making factors (e.g. peers, access and location). The trait‐like stability of alcohol consumption is generally maintained into and throughout adulthood [47, 48].

When we examined the reciprocal relationships between ARA and HED, it was past HED that shaped future attitudes rather than attitudes driving behaviours. Within adolescents, ARA appear to lag HED. This lag has also been observed in prior alcohol [21] and smoking studies [49]. These findings are much more consistent with a cognitive dissonance and rationalization model of the relationship between ARA and drinking behaviour than theoretical models that proposed attitudes as the precursors of behaviours [21].

With non‐drinkers, it is likely that drinking status remains highly consistent with ARA until the transition to HED, when attitudes then evolve to support the newly acquired drinking status (i.e. become more pro‐alcohol). Therefore, negative attitudes are likely to support continued negative attitudes towards alcohol (via the autoregressive pathway), until situational factors create an environment conducive to a change in drinking status (engaging in HED). The resultant cognitive dissonance arising from the mismatch between ARA and HED appears more likely to be resolved by adopting more pro‐alcohol attitudes than desisting from future HED (as HED demonstrates strong autoregressive effects within the model). Any causal association between these two processes appears predominantly in the direction of drinking behaviour impacting upon ARA, and not the reverse. This finding is in stark contrast to the body of existing research supporting models such as the Theory of Planned Behaviour that propose causal attitudes to behaviour relationships [8, 50]. However, the majority of studies testing models such as the Theory of Planned Behaviour have failed to include reciprocal paths for the influence of behaviour on changes in attitudes and intentions, as was modelled here (see, for example, [5, 11, 51). These two processes are developmentally intertwined, but prior research has tended to view the inter‐relationship from one direction only, with ARA the predictor and drinking behaviour the outcome. Permitting the relationship between ARA and behaviour to be cross‐lagged appears to fundamentally change the observed relationships, where drinking behaviour is the predictor of ARA.

It is worth noting that, in addition to the observed autoregressive and cross‐lagged effects, ARA and HED were both shaped by the wider school environment inhabited by young people. In particular, a higher level of poverty within the school (as indexed by the proportion of pupils on free school meals) was associated with more positive ARA and higher HED. This relationship between school‐level SES and alcohol consumption has been noted in some [52] but not all research [53, 54]. School‐level HED was also higher within schools in Scotland and within mixed‐sex schools. These effects may be due in part to the peer drinking cultures established within certain types of school [52, 55].

Given the literature described in the Introduction, it is unsurprising that reducing positive attitudes towards alcohol is a significant component in many alcohol prevention interventions aimed at adolescents [56, 57]. However, as evidenced in this study, the relationships between ARA and alcohol‐related behavioural outcomes are complex. Evaluations of many alcohol education programmes have shown larger impacts upon attitudes than on drinking behaviours [57, 58]. However, studies undertaking mediation analysis of trial outcomes have failed to show ARA as a significant mediator of intervention effects on HED or life‐time drinking [59, 60, 61]. Unlike the STAMPP cRCT outcome analysis [28, 30], that detected a significant intervention effect on HED, this analysis only found differences across the study arms for ARA, and not HED. While the RI‐CLPM presented here was not specified to be a definitive test of intervention effects, and clearly demonstrates that changes in HED precede changes in ARA, this finding is interesting and warrants future consideration. Additional exploratory probing of the effect of the cRCT on the unfolding relationship between attitudes related to alcohol and heavy episodic drinking may provide additional insights into the effects of the cRCT beyond the scope of the current paper.

While it is hard to see future prevention interventions not attempting to reduce positive ARA among pupils, together with attempting to reduce their engagement in HED and subsequent exposure to alcohol‐related harms, there is little evidence presented here to support ARA being considered as either a relevant secondary outcome (together with primary consumption outcomes such as HED) within an evaluation trial or as a key mediator of behavioural outcomes within an intervention logic model.

While the study has several strengths, including a large sample size and multiple time‐points, several limitations must also be acknowledged. The data are self‐reported, therefore may be subject to reporting biases. While measurement error associated with adolescent alcohol use is considerably less than that seen for other substances [62, 63], such biases could attenuate cross‐lagged effects [64]. The sample is also subject to a loss of respondents due to attrition. In particular, attrition rates were higher among those students who were male, reported early‐onset drinking and had lower socio‐economic status. As all three characteristics are known to be associated with an increased risk of HED [65], we can assume that attrition was higher among those pupils most likely to engage in HED and to have more positive attitudes to alcohol. While the modelling strategy assumes that missing data due to attrition are conditionally missing at random (i.e. are systematically related to observed covariates already within the model), we recognize that it is impossible to rule out that the missing data are missing not at random (i.e. is systematically related to unobserved covariates). While the Bayes estimator handles missing at random in the similar manner to full information maximum likelihood [66] we are not aware, as yet, of any direct application of recognized missing not at random strategies (see [67]) within a RI‐CLPM framework using a Bayes estimator.

Even though the sample is largely representative of the population of adolescents from which it is drawn, the findings may not generalize to other jurisdictions or other populations. For example, the relatively low levels of ethnic/racial diversity within the sample may not be found in other adolescent cohorts or cultures.

While the RI‐CLPM approach has been successfully implemented in the field of alcohol studies (see, for example [34, 68, 69]) it is not without potential limitations, particularly in the presence of unmeasured confounding variables [70]. However, potential solutions have been proposed, including the use of an additional lag (i.e. cross‐lag 2‐panel model) to control for unobserved confounding [71]. It has also been argued that RI‐CLPM may not be the most appropriated modelling strategy for unpacking within‐ and between‐individual differences in cross‐lagged relationships when the processes under study have not achieved equilibrium, such as when there is inherent change within the processes as part of normal development or due to the impact of an intervention [72], as is the case here. The added complexity of RI‐CLPM, relative to other crossed‐lagged approaches, means that RI‐CLPM require more data (larger sample and more sweeps) to produce robust replicable estimates [73]. While this study has a large sample size it is restricted to only four data sweeps.

Finally, the estimated models did not include contemporaneous reciprocal relationships between ARA and HED (e.g. ARA3 → HED3). It is possible that the time lag between ARA and HED is much shorter that the cross‐lags modelled here. However, RI‐CLPM incorporating reciprocal effects are only beginning to emerge and further work in this area is needed (for further discussion see [74]).

Notwithstanding these limitations, the study findings have significant implications for prevention theory, and also the design and evaluation of alcohol prevention interventions. They challenge the commonly held belief that attitudes towards alcohol drive alcohol use behaviours. On the contrary, the results herein suggest that attitudes are much more of a product of behaviour than vice versa. Interventions aiming to prevent adolescent risk‐taking through promoting negative attitudes towards the behaviour may be misguided. Future theory and practice should reflect the observed temporal relationship between alcohol attitudes and drinking behaviour.

## AUTHOR CONTRIBUTIONS

**Andrew Percy:** Conceptualization (equal); data curation (equal); formal analysis (equal); funding acquisition (equal); methodology (equal); writing–original draft (lead); writing–review and editing (lead). **R. Noah Padgett:** Formal analysis (equal); investigation (equal); writing–original draft (equal); writing–review and editing (equal). **Michael T. McKay:** Conceptualization (equal); data curation (equal); funding acquisition (equal); investigation (equal); methodology (equal); project administration (equal); writing–original draft (equal); writing–review and editing (equal). **Jon C. Cole:** Conceptualization (equal); funding acquisition (equal); investigation (equal); methodology (equal); writing–original draft (equal); writing–review and editing (equal). **Gregor Burkhart:** Conceptualization (equal); writing–original draft (equal). **Chloe Brennan:** writing–review and editing (equal). **Harry R. Sumnall:** Conceptualization (equal); funding acquisition (equal); investigation (equal); methodology (equal); project administration (equal); writing–original draft (equal).

## DECLARATION OF INTERESTS

The sponsor university (LJMU) for the original STAMPP trial received and administered a payment from the alcohol industry for printing of student workbooks in the Glasgow trial site only. A.P. reports that he has previously received funding from the European Foundation of Alcohol Research (ERAB) in relation to the development of statistical models for longitudinal data (2008–10). H.S. reports that his department has previously received funding from the alcohol industry (indirectly via the industry funded Drinkaware charity) for unrelated primary research.

## TRIAL REGISTRATION

ISRCTN47028486 (http://www.isrctn.com/ISRCTN47028486). The date of trial registration was 23/09/2011, and school recruitment began 01/11/2011.

## Supporting information


**Table S1.** Supplemental model specification summary.
**Table S2.** Autoregressive and Cross‐Lagged parameter estimates across primary and supplementary models.
**Figure S1**. *Frequency was significantly predictive of attitudes*.
**Figure S2**. *Time invariant covariates were predictive of frequency than attitudes*.
**Figure S3**. A*lcohol related harms was significantly predictive of attitudes*.
**Figure S4**. *Time invariant covariates were predictive of alcohol related harms than attitudes*.
**Table S3:** ARA Factor Loadings (1 factor solution).

## Data Availability

The data that support the findings of this study are openly available in the Queen's University Belfast data repository, https://doi.org/10.17034/498b761c-0051-45c0-a92b-4398f8fa501c. Full details of the Mplus code used in the analysis can be found at https://osf.io/bmhak/?view_only=67d2dc1013af46a880662a2c8067635a.
