# Supplementary material for: Disentangling the temporal relationship between alcohol‐related attitudes and heavy episodic drinking in adolescents within a randomized controlled trial
Source: Addiction. 2024 Dec 10;120(4):645–54. doi: 10.1111/add.16721 (PMC11907331; doi:10.1111/add.16721)
Supplement: Supplementary file 1 — Table S1. Supplemental model specification summary. Table S2. Autoregressive and Cross‐Lagged parameter estimates across primary and supplementary models. Figure S1. Frequency was significantly predictive of attitudes. Figure S2. Time invariant covariates were predictive of frequency than attitudes. Figure S3. Alcohol related harms was significantly predictive of attitudes. Figure S4. Time invariant covariates were predictive of alcohol related harms than attitudes. Table S3: ARA Factor Loadings (1 factor solution). [file ADD-120-645-s001.docx]

**Supplemental Material**

**OSF Repository**

[*https://osf.io/bmhak/?view_only=67d2dc1013af46a880662a2c8067635a*](https://osf.io/bmhak/?view_only=67d2dc1013af46a880662a2c8067635a)

**Supplementary modelling details**

The Bayes estimator in Mplus was used within the primary model to generate Bayesian posterior parameter estimates with credibility intervals. While ML provides a point estimate for model parameters with symmetrical confidence intervals, the Bayes estimator within Mplus estimates a posterior distribution for each parameter. The median of this posterior distribution was used to obtain a point estimate for model parameters. Bayesian credibility intervals were based on the percentiles of the posterior distribution (in this case the 2.5% quantile or 97.5% quantile) allowing for skewed distributions. As diffuse (default) priors were used, ML and Bayes results would be expected to be close given the large sample used in this analysis.

For the Bayesian estimation, 10,000 iterations were run across 4 chains. The first 5000 iterations per chain were discarded as burn-in. Each posterior distribution was evaluated for convergence using the Gelman-Rubin-Brooks convergence criterion, where values closer to 1 indicate convergence.

**Sensitivity Tests**

*Estimator*: The primary model (RI-CLPM with Bayes estimator) was also estimated using the WLSMV estimator to test the sensitivity of the model to the specific estimator used.

*Alcohol measures:* Models were estimated incorporating different alcohol measures (frequency of drinking alcohol and personal experiences of alcohol related harms) to test the sensitivity of models to the measurement of drinking behaviour. Full details of these alternative measures can be found below.

*Attitude measure*: Exploratory factor analysis of the six items of the attitudes towards alcohol measure suggested that two of the six items loaded weakly on the single factor. Models were estimated with both the six-item scale and a revised four-item scale (dropping the two lowest loading items). The EFA results are included below.

*Misspecification of longitudinal mechanisms:* To test the sensitivity of the inferences made using the RI-CLPM to misspecifications of the longitudinal mechanism, a range of additional models were estimated, including standard CLPM, models with additional lags, models with constraints on the autoregressive and cross-lagged effects, and models with cluster robust standard errors accounting for clustering at the school rather than school level random intercepts.

No substantively meaningful differences were detected in results among the various models estimated. Full details of the various models estimated and the different measures of attitudes and alcohol consumption can be found in the supplementary material.

| **Table S1.** Supplemental model specification summary | | | | | | |
| --- | --- | --- | --- | --- | --- | --- |
| Model | Model  Type | Estimation  Method | Cluster  Robust | Lag | Missing  Data | Report in  Text |
| 1. CLPM | Observed | Bayes | No | Lag-1 | Model-Imputed |  |
| 2. CLPM | Observed | WLSMV | Yes | Lag-1 | Pairwise complete |  |
| 3. CLPM | Observed | Bayes | No | Lag-2 | Model-Imputed |  |
| 4. CLPM | Observed | WLSMV | Yes | Lag-2 | Pairwise complete |  |
| 5. RI-CLPM | Latent | Bayes | No | Lag-1 | Model-Imputed |  |
| 6. RI-CLPM | Latent | WLSMV | Yes | Lag-1 | Pairwise complete |  |
| 7. RI-CLPM | Latent | Bayes | No | Lag-2 | Model-Imputed |  |
| 8. RI-CLPM | Latent | WLSMV | Yes | Lag-2 | Pairwise complete |  |
| **9. Multilevel RI-CLPM** | **Latent** | **Bayes** | **Yes*** | **Lag-1** | **Model-Imputed** | **Yes** |
| 10. Multilevel RI-CLPM | Latent | WLSMV | Yes | Lag-1 | Pairwise complete |  |
| 11. Multilevel RI-CLPM | Latent | Bayes | Yes* | Lag-2 | Model-Imputed |  |
| 12. Multilevel RI-CLPM | Latent | WLSMV | Yes | Lag-2 | Pairwise complete |  |
| 13. Multilevel RI-CLPM  *(Drinking Frequency)* | Latent | Bayes | Yes* | Lag-1 | Model-Imputed |  |
| 14. Multilevel RI-CLPM  *(Alcohol harms)* | Latent | Bayes | Yes* | Lag-1 | Model-Imputed |  |
| 15. Multilevel RI-CLPM  *(4 item Attitude measure)* | Latent | Bayes | Yes* | Lag-1 | Model-Imputed |  |

Note. *The multilevel RI-CLPM is cluster robust as the cluster effect (pupils in schools) is directly modeled.

| Table S2: Autoregressive and Cross-Lagged parameter estimates across primary and supplementary models | | | | | | | | | | | | |
| --- | --- | --- | --- | --- | --- | --- | --- | --- | --- | --- | --- | --- |
|  | Autoregressive effects | | | | | | Cross lag effects | | | | | |
| Model Number | HED1 → HED2 | HED2 → HED3 | HED3 → HED4 | ARA1 → ARA2 | ARA2 → ARA3 | ARA3 → ARA4 | HED1 → ARA2 | HED2 → ARA3 | HED3 → ARA4 | ARA1 → HED2 | ARA2 → HED3 | ARA3 → HED4 |
| 1 | 0.64 | 0.73 | 0.74 | 0.28 | 0.34 | 0.38 | 0.07 | 0.13 | 0.18 | 0.03 | -0.02 | -0.06 |
| 2 | 0.34 | 0.71 | 0.75 | 0.34 | 0.39 | 0.48 | 0.04 | 0.13 | 0.14 | 0.10 | -0.02 | -0.07 |
| 3 | 0.60 | 0.56 | 0.61 | 0.28 | 0.30 | 0.30 | 0.07 | 0.05 | 0.14 | 0.02 | -0.04 | -0.04 |
| 4 | 0.27 | 0.66 | 0.62 | 0.29 | 0.29 | 0.30 | 0.04 | 0.09 | 0.18 | 0.05 | -0.08 | -0.05 |
| 5 | 0.37 | 0.43 | 0.38 | 0.07 | 0.11 | 0.17 | 0.07 | 0.23 | 0.25 | 0.03 | 0.00 | -0.03 |
| 6 | 0.59 | 0.82 | 0.83 | 0.09 | 0.17 | 0.21 | 0.05 | 0.18 | 0.24 | 0.04 | -0.09 | -0.10 |
| 7 | 0.40 | 0.38 | 0.42 | 0.11 | 0.16 | 0.19 | 0.10 | 0.18 | 0.18 | 0.05 | -0.01 | -0.03 |
| 8 | 0.69 | 0.81 | 0.90 | 0.14 | 0.19 | 0.21 | 0.08 | 0.19 | 0.20 | 0.05 | -0.09 | -0.11 |
| **9 Primary** | **0.30** | **0.35** | **0.39** | **0.07** | **0.13** | **0.16** | **0.03** | **0.13** | **0.23** | **0.01** | **-0.01** | **-0.02** |
| 10 | 0.32 | 0.36 | 0.37 | 0.07 | 0.14 | 0.17 | 0.06 | 0.15 | 0.24 | 0.00 | 0.00 | -0.03 |
| 11 | 0.38 | 0.35 | 0.45 | 0.11 | 0.18 | 0.19 | 0.04 | 0.07 | 0.15 | 0.01 | -0.03 | -0.03 |
| 12 | 0.44 | 0.40 | 0.47 | 0.12 | 0.19 | 0.19 | 0.05 | 0.06 | 0.14 | -0.01 | -0.06 | -0.05 |
| 13 | 0.22 | 0.24 | 0.28 | 0.06 | 0.14 | 0.21 | -0.01 | 0.04 | 0.12 | -0.02 | 0.02 | 0.01 |
| 14 | 0.34 | 0.40 | 0.42 | 0.07 | 0.13 | 0.17 | 0.07 | 0.14 | 0.25 | 0.02 | 0.01 | -0.03 |
| 15 | 0.31 | 0.36 | 0.39 | 0.09 | 0.13 | 0.16 | 0.05 | 0.14 | 0.27 | 0.01 | -0.01 | -0.01 |

Notes: See table S1 for full specification for each estimated model

**Supplementary Figures (S1-S4)**


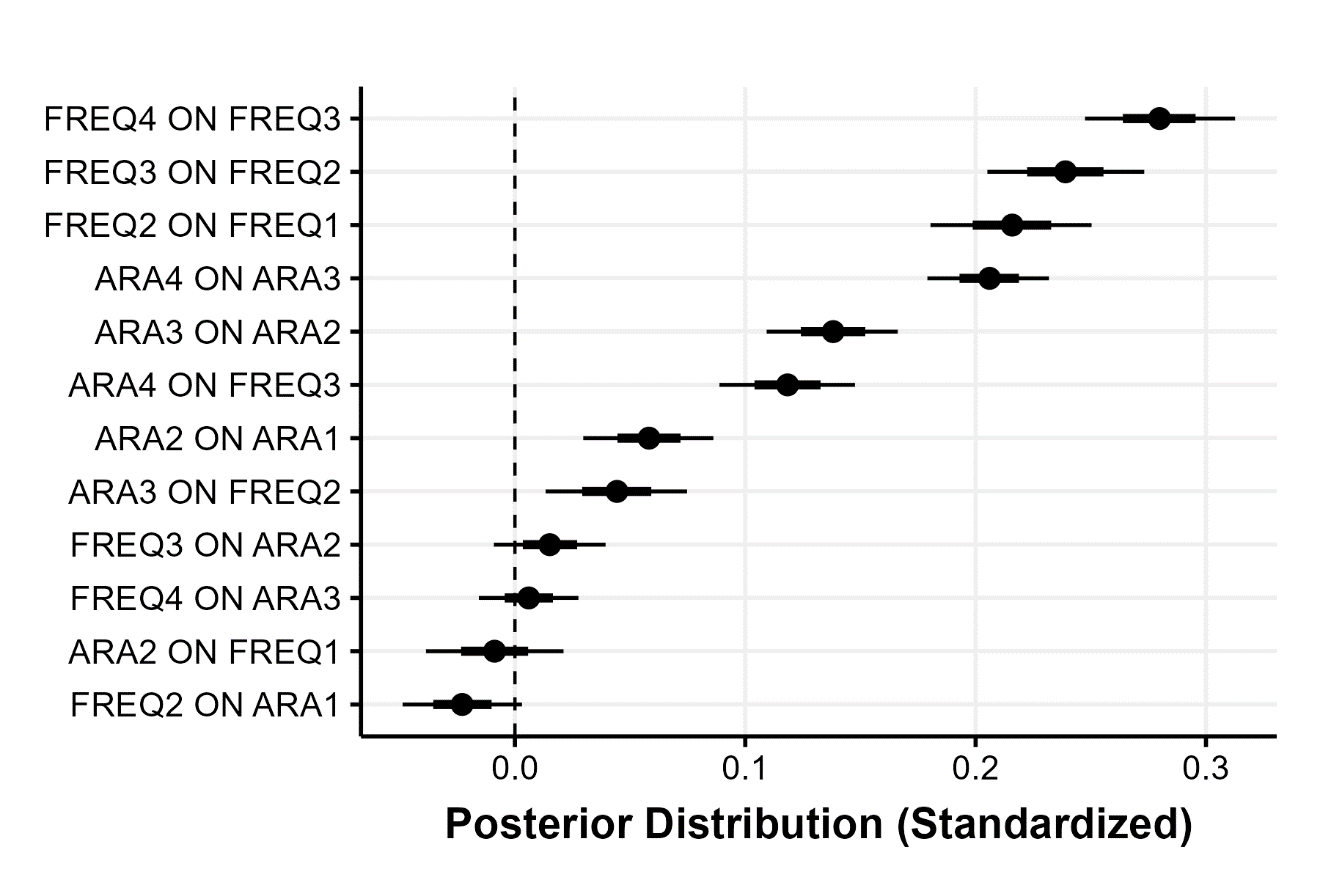


**Figure S1**. *Frequency was significantly predictive of attitudes.*

*Note*. Figure depicts the 95% central probability of posterior distributions.

  
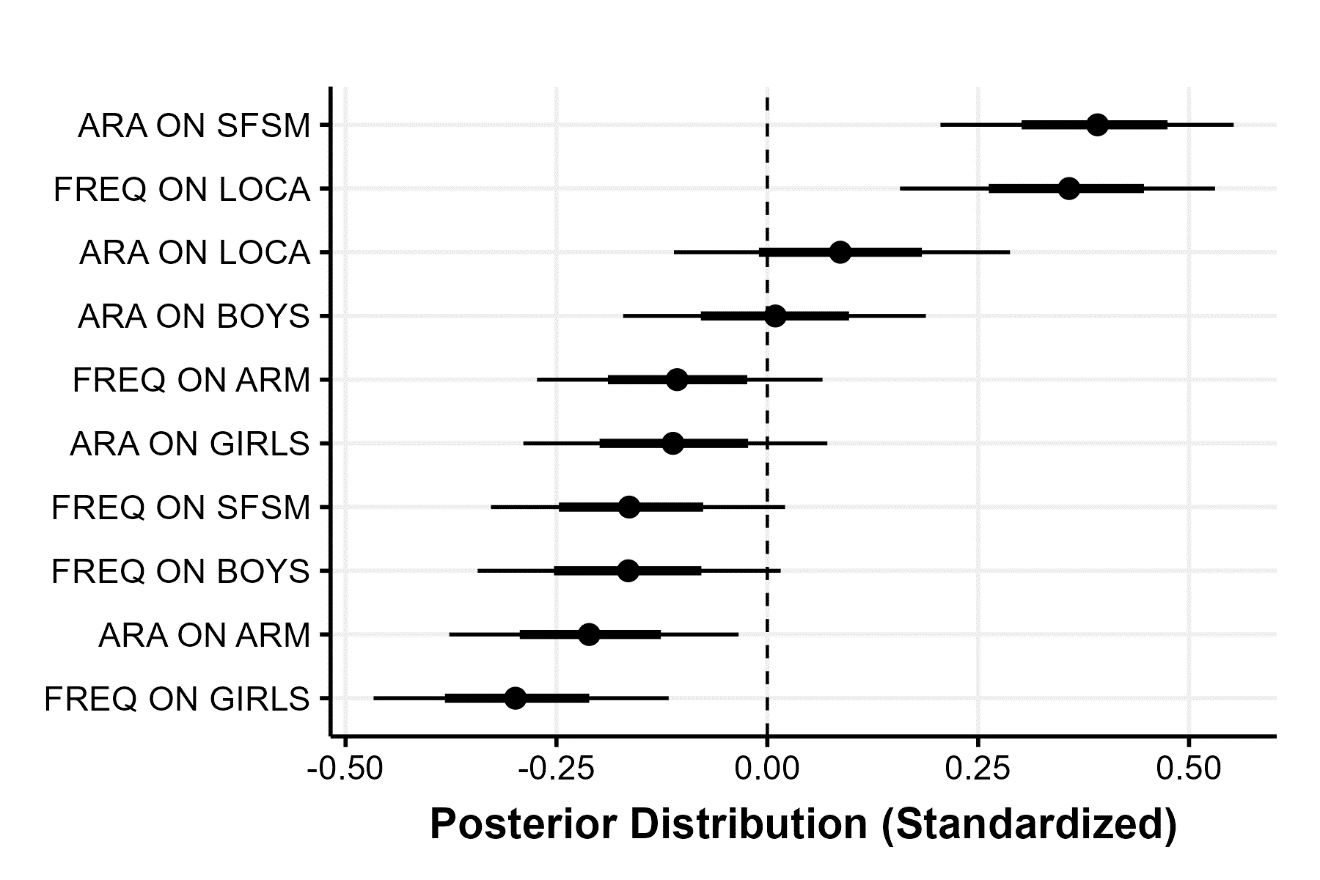


**Figure S2**. *Time invariant covariates were predictive of frequency than attitudes.*

*Note.* SFSM = Socioeconomic status (tertile split); LOCA = location of school; BOYS = all-boys school; GIRLS = all-girls school; and ARM = study arm.

**
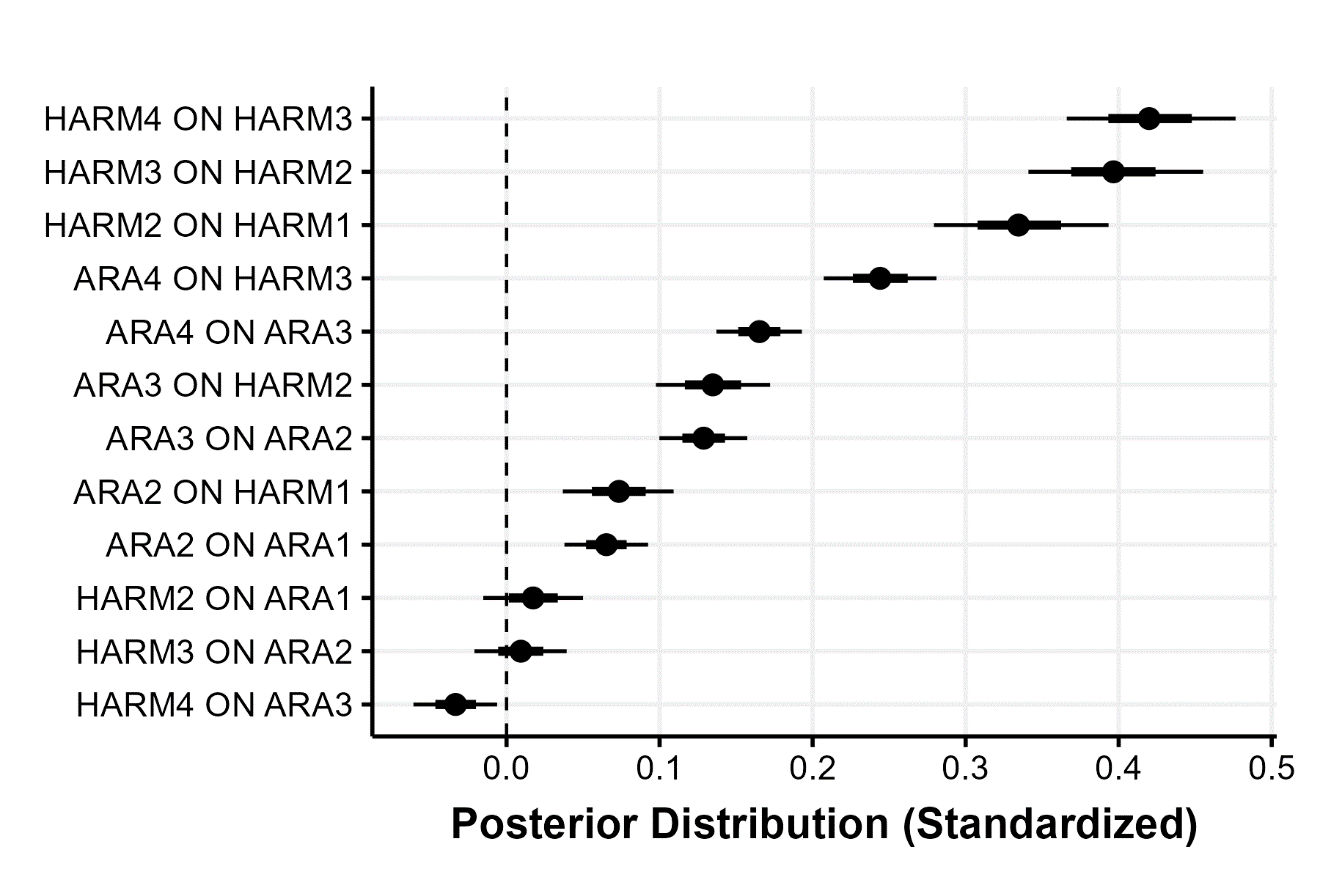
**

**Figure S3**. A*lcohol related harms was significantly predictive of attitudes.*

*Note*. Figure depicts the 95% central probability of posterior distributions.


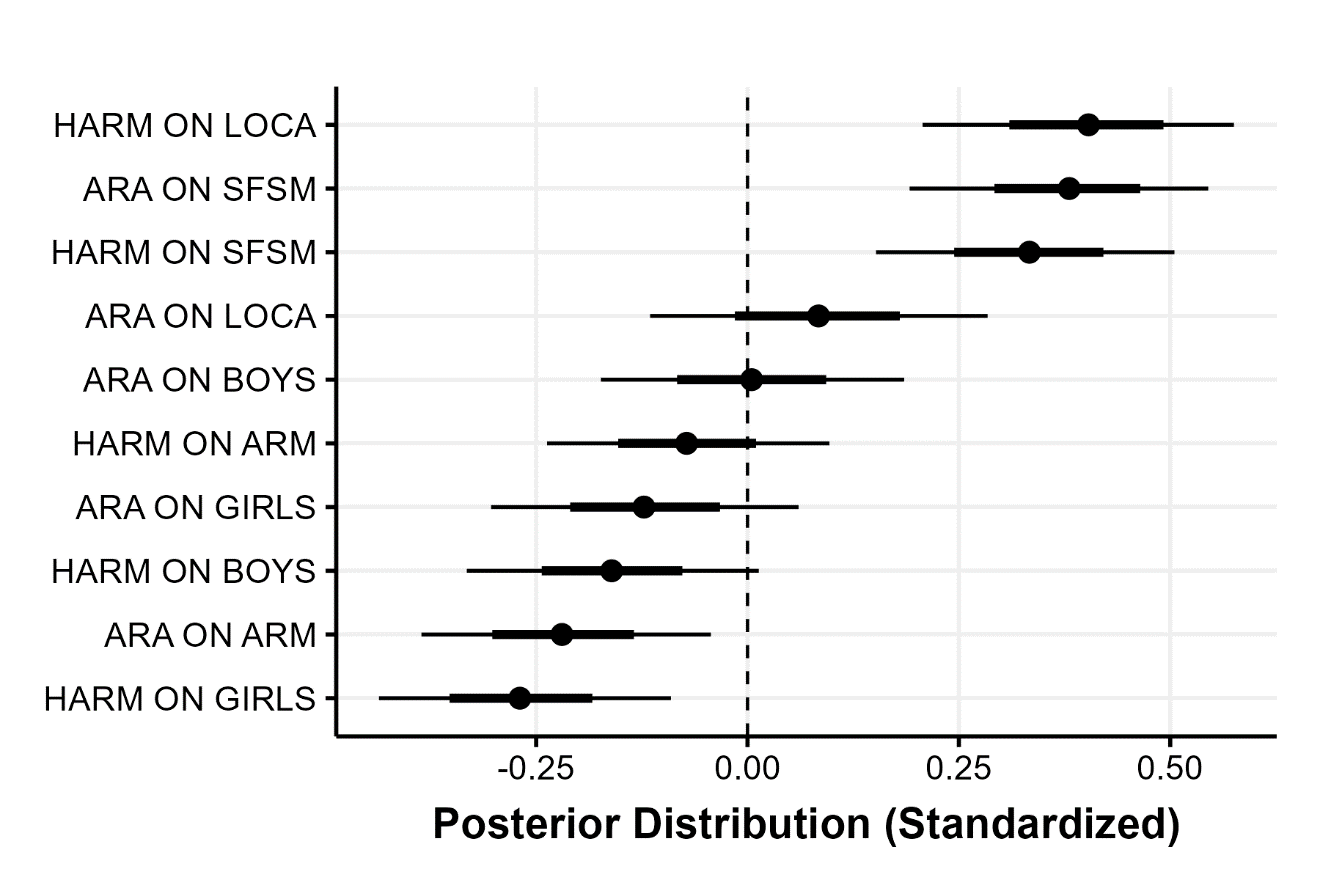


**Figure S4**. *Time invariant covariates were predictive of alcohol related harms than attitudes.*

*Note.* SFSM = Socioeconomic status (tertile split); LOCA = location of school; BOYS = all-boys school; GIRLS = all-girls school; and ARM = study arm.  

**Alternative measures used in the sensitivity tests**

**Alcohol-related attitudes (6 item and 4 item versions)**

1. I would accept a lift from a person who had been drinking a lot

2. I know a lot about alcohol*

3. People my age have a good time at parties when they get drunk†

4. It is OK for young people to drink as long as they do it safely*†

5. Young people can enjoy alcohol without getting drunk*†

6. Sometimes I plan to get drunk†

* Items reverse coded in the final summed attitude score.

† Items included in the 4 item version of the scale

**(each item scored on a 5-point Likert scale, *Strongly disagree* – *Strongly agree*)**

**Drinking Frequency**

Each year pupils were asked “How frequently do you drinking alcohol?” Answer categories were: never (0), <monthly (1), once per month (2), every 2 weeks (3), once per week (4), 2/3 times per week (5), every day (6).

**Alcohol related harms**

Pupils were asked about their experiences of 16 different types of alcohol related harm caused by their own drinking in the last six months. Responses were initially summed then dichotomised (0 = no harms reported / 1 = any harms reported). The full list is below:

In the last six months……

1. Have you planned to get drunk?
2. Did you drink more than planned to?
3. Were sick after you had been drinking?
4. Did you have a hangover after you had been drinking?
5. Were unable to remember things that had happened when you had been drinking?
6. Did you verbally abuse someone when you had been drinking?
7. Did you get into a physical fight when you had been drinking?
8. Did you damage property when you had been drinking?
9. Were you sexually harassed when you had been drinking?
10. Was your school performance affected (e.g., day off) because you had been drinking?
11. Did you get in trouble with friends because of your drinking?
12. Did you get in trouble with a boyfriend/girlfriend because of your drinking?
13. Did you get in trouble with your parents because of your drinking?
14. Did you get in trouble at school because of your drinking?
15. Did you get in trouble with the Police because of drinking?
16. Did you have to attend a doctor or hospital because of your own drinking?

**Alcohol Related Attitudes (ARA) EFA Factor Loadings**

The estimated coefficient alpha for ARA was 0.59.

| **Table S3:** ARA Factor Loadings (1 factor solution) | |
| --- | --- |
| Item | Factor loadings |
| ARA1 | .236 |
| ARA2* | .317 |
| ARA3 † | .662 |
| ARA4*† | .739 |
| ARA5*† | .423 |
| ARA6 † | .633 |
| Extraction Method: Maximum Likelihood. | |
| Chi-Square: 1125.961; df: 9; p<0.001 | |
| * Items reverse coded in the final summed attitude score.  † Items included in the 4 item version of the scale | |
